# Allegheny County Medical Society

**Published:** 1891-07

**Authors:** 


### Society Reports.

### ALLEGHENY COUNTY MEDICAL SOCIETY.

### Scientific Meeting, May 19th, 1891.

C. S. Shaw, M. D., Vice-President, in the chair.

The topic for discussion, "Tuberculosis," was opened by Dr. R. W. Stewart. See page 265.

Dr. T. C. Christy : I think the paper of Dr. Stewart concisely and accurately written. His experience is of value to all of us. I agree with him on his points on the physical diagnosis of tuberculosis. I believe that every physician is in duty bound to strip his patient and examine thoroughly ; it is the only true way to examine a case. It seems to me that there is no better anti-bacillic material than pure blood ; that is, there is nothing that will destroy bacilli so much as pure blood. Secondly, that the larger cells that we find in the blood vessels will destroy the germs, and keeping these two points in view, we have a basis of treatment which cannot be surpassed. We must follow the line of Dr. Stewart's plan of treatment, that it is pure air, hygienic surroundings and care that are most efficacious in treating this disease. Now, going back to the idea that pure blood is the best germicide we have, anything that will induce the formation of pure blood will place these patients in their early years in a much safer condition than they would otherwise be ; that is, in public school buildings and in their daily exercise we can do much to prevent them from contracting disease. Another point which has been lately advanced by a French writer: He says that animals are more easily affected by bacillic injections where they have been deprived of food ; or, in other words, that hunger will make persons more liable to contract this disease. Taking pigeons, which are not particularly susceptible to bacillic influence when deprived of food for several days, injections of the bacilli caused inoculation and death, whereas, where they were well fed, they were free from the influence of the bacilli. I think if we are careful in the examination of patients, careful in the management of their case not to underfeed them and to look after their hygienic surroundings, we accomplish more than we can in any other direction.

DR. GRUBE: One person in seven dies of tubecrulosis, and I venture to say that one-half of the race have the germs of tuberculosis in their system. It is natural that a disease of such importance should command our careful attention. Our profession is exceedingly active in devising some means for the treatment of this disease. I think the greatest point, however, one which the speaker who has just preceded me brought up, is the early diagnosis and treatment of this disease. It is a disease of predisposition, and I believe that the ground work is laid in the embryo. I think here is the secret of the predisposition to tuberculosis. Dr. Trudeau, of New York, made some valuable experiments. He made an experiment with which doubtless many of you are familiar, and which will, I believe, become classic. He took fifteen rabbits and divided them into three lots. With the first five, he injected the pure solution of bacilli into their lungs, into the abdominal cavity, and under the skin of the neck. These five he placed in a dark cellar. The next five were inoculated in a like manner, and were turned loose in an island on the lake in the month of June, and they were fed with vegetables in addition to the vegetation upon the island which was accessible. The other five were not inoculated at all, but a hole was dug in the middle of a field; this hole was ten feet in depth and the remaining five rabbits, having been placed in the box, were lowered to the bottom of the pit. The box was covered with boards and dirt, a trap hole being left for the introduction of food. Of the first five rabbits, in the cellar, four died within a month, and all of general tubercular infection. This existed not only in the lungs but generally. The five in the pit were taken out perfectly well; they were emaciated, their coats were rough, but there were no signs of tuberculosis in any of them. Of the five which were liberated on the island, one died within a month; it had some tubercular affection of the lungs. The other four grew fat, and at the end of four months, were perfectly well and in fine condition; they were killed then, and not even the point of inoculation could be found; there was no sign of tuberculosis whatever. Now I think this is a lesson which can be made of practical use. Some one has said that the cause of consumption in London was that the poor were too poor to buy butter and fat, and the rich injured their digestion with two much pastry. What I want to bring out is the point that the rapid loss of weight in one who has the predisposition ought at once to call our attention to the fact that he is preparing the way for bacilli, and it is an open question whether in such cases the loss of weight is caused by bacilli or whether the ground is merely being prepared for them; I am inclined to believe that the latter is the case. Of course with a great many patients it is impossible to do very much. It is a matter of ability to bring the patient through the early stages of the disease safely. This means giving up a vocation which they cannot afford to give up. The question of occupation is one of the principal ones in this disease. Nearly one-half the printers die of consumption.

DR. BUCHANAN: Dr. Grube's remarks call to my mind the theory proposed by that excellent pathologist, Dr. Formad, to the effect that tubercular individuals were such as had narrow lymph spaces, that it was the obstruction in the lymph spaces that caused tuberculosis, and he endeavored to prove this by microscopical measurements of the lymph spaces in different species of animals. I believe he made the same observations on human beings who were tuberculous, although I am not sure of that. I do not know whether Dr. Formad has endeavored to reconcile his theories with the modern views of tuberculosis as brought forward by Koch, but I believe that Dr. Formad's theory had its origin about the time that Prof. Koch discovered the bacilli of tuberculosis.

DR. BANE: I have been in Colorado several weeks, and there met many people who had gone to that State on account of consumption, who went there expecting to die, but whose lives have been spared by reason of the climate, or you might say, by the air, which is dry and light, especially when you go where it is so high as it is at Colorado Springs and Denver. As has been brought out by Dr. Stewart, in order to get sufficient oxygen more air is inhaled, the lungs are expanded and the cells which are not used in this district are employed there, and there is no doubt this is one of the reasons why those patients who have not a sufficient breathing space are so much better in that climate. It is a fact that many persons die in Colorado of consumption, but unfortunately they go there too late to be benefited ; if they would go while in the incipient stages, certainly many of them would live to a good old age, but as a last resort many go there when they should stay at home, when they would live longer at home surrounded by home influences, than they do in the climate of Colorado, with all it advantages. There are many things to be said about sending a patient to Colorado. Persons suffering from heart disease fare badly in the climate on account of the rarity of the atmosphere ; persons of nervous temperament do not rest well there. I met there a friend, a neighbor of mine in the profession, who some six years ago went to Colorado Springs. He was told that it was not worth his while to go, that he would not live more than six months. However, he went, and began to improve from the time he reached Colorado. When he arrived there, his temperature dropped down to normal and there remained. He continued to cough for eighteen months. Then he thought he might come home to Philadelphia ; he returned home, and after remaining some two weeks, his cough returned and he was obliged to once more go to Colorado, and on his reaching that place the second time it took him as long as before to become free from the cough and feel well again. He then learned he would have to remain in Colorado if he wished to live long. From Colorado Springs he went to Denver, where he has a good practice and good health. From his appearance I should think he weighs about 200 pounds. One physcian who went there with consumption now weighs 300 pounds. Certainly we have patients here that we cannot expect to cure in this climate that we are not justified in keeping here if they have the means to take them to a climate such as exists in Colorado. This is the impression made upon me after visiting the State and meeting these people. True, they have to remain there, but it is no punishment to live in Colorado, where they have as fine a climate as I have ever seen, and where eastern people, with eastern business and social ideas, form the bulk of the population.

DR. RIGG: I was much pleased with Dr. Stewart's paper. I think it deserves our careful consideration, and there are many points in it that would bear further investigation. The doctor's calling attention to early diagnosis is a very important thing, also, the natural tendency or the inherited condition. The doctor stated that tuberculosis is not an inherited disease, which fact is well established at this time. The predisposition, however, is inherited. We have it in the animal as well as in the human family. You take the rabbit and the Guinea pig, they are naturally tuberculous; the dog and cat are naturally non-tuberculous; the rat is not naturally tuberculous. Now, you take a family of children coming into the school-room, and it is a comparatively easy matter to see which are tuberculous children. The conditions under which they have been reared may have had much to do with developing the condition. I am not sure that nationality has not something to do with it. Six or seven years ago, I made some little investigation on that line. This investigation of mine was conducted through the southern part of Westmoreland county, and the northern part of Fayette, and covered a period of three years. I suppose in all I collected statistics from 150 different families. In regard to the investigation of Dr. Formad, it was my pleasure to be with him a part of the time he was making the investigation. He took the children in the hospital and held *post-mortems* there and examined some of the tissue from all children who were believed to be tuberculous yet had sickened and died of acute diseases, and also from children who had died of tuberculosis. He examined the connective tissue from other children that had died that we believed to be non-tuberculous, and there was a marked change, a marked difference in the lymph channels that fully convinced me that there was more in it than mere theory.

My recollection is that he had modified his views at that time greatly with reference to the cause of the bacilli. He admitted that the bacilli might be the carrier of tuberculosis, and that it might possibly be the specific poison.

The question not settled in my mind is this: are the bacilli, as a rule, one of the early evidences of tuberculosis? Take a subject that is predisposed to it, that has, if you please, the narrow lymph channels, and will you not have a certain amount of engorgement in the lungs before the bacilli? In other words, are the bacilli present before there is inflammation, or are the bacilli formed subsequently to the inflammation? In regard to the influence of surroundings that has been referred to by both the gentlemen who have spoken. An experiment was made on rats, I do not recall the particular poison that was used, but it made an impression on my mind. A number of rats were caught and kept under favorable conditions; they were injected with specific poison and turned loose in comfortable circumstances under hygienic surroundings. Others were put in a treadmill, where they were obliged to work and kept at it until they were tired. They sickened and died with tuberculous disease. The first lot lived and thrived. But that is only one evidence of many that pure air, pure food and pure surroundings fortify the system against the attack of any disease; I do not care whether it is tuberculosis or something else; if there is a special tendency to tuberculosis, it will likely be that; if there is a tendency to something else, it will likely be that. In regard to the influence of a rarefied atmosphere; I think there is a good deal in that, and we might see why, if we bear in mind the fact, that the apex of the lungs is the portion usually involved first. It is, perhaps, the portion of the lung least used under ordinary circumstances; expansion is less there than at any other portion of the lung.

I have nothing to say as to treatment. In regard to the tuberculin, it seems to me that the theory on which it is given will hardly bear us out in using it constantly. I think it a step in the right direction, yet the question that comes up in my mind is whether or not you can generate a poison from the bacilli that will do what is claimed for the lymph, or that will aid nature in throwing off the diseased tissue. It seems to me we cannot expect much on that theory. As far as I am personally concerned, I want to see some more rational theory advanced or else more satisfactory experience. I have two cases on hand now, and for a month I have debated in my mind whether or not to advise a trial, and I have not had the courage to do it. The patients are willing and ready to take the risks if so advised, and if there can be anything that will help me to decide, I would be very glad. One of them is a case of tubercular trouble, pulmonary tuberculosis, and the other is a case which Dr. Buchanan saw with me six weeks ago, tuberculosis of the lymphatic glands and the ankle joint.

DR. STEWART: As to the doctor's remarks in regard to Colorado. A patient came to my office from Colorado recently; he had returned to this city and gone to work. I examined his lungs and found marked consolidation of the upper portion of both lungs. I told him to go back to Denver as soon as he could. I do not think I could have done him any good. With regard to Dr. Rigg's question as to whether the bacilli are present at the early stage, my understanding is that the presence of the bacilli is an essential factor at the initial stage of the disease, and that the tubercular products are the result of the growth of the bacilli.

I would speak a few words of my own results in the use of Koch's lymph. The first case was for diagnostic purposes on a patient in the Mercy Hospital, but he left before a result could be ascertained. The second and third cases were in St. Francis Hospital; both cases were confined to their rooms and the disease was progressing rapidly; both improved at once and have since left the hospital, although still continuing under treatment, and their improvement still continues. The remaining cases were treated in Mercy Hospital; one advanced consumptive did not improve and has since died. Another remained two weeks in the hospital with but slight improvement, but continues the treatment at his home, and is steadily improving. A case of destruction of the nose supposed to be lupus, did not give any reaction even to a dose of twenty-six milligrammes, and was not benefited. I considered the condition as that of an epithelioma. A case of lupus of eyelid at present under treatment gives promise of a cure.

HEMORRHAGE AFTER LITHOTOMY.

DR. J. W. MACFARLANE: On the 15th of last April, assisted by Dr. Small and the residents of the Western Pennsylvania Hospital, I did a left lateral lithotomy upon Jas. Mehen, removing a mulberry calculus weighing six drachms.

The history of the case is as follows: The lad is sixteen years of age and undersized. His trouble dates back to last July, when he moved to, and secured employment in, Kittanning. The pain and vesical irritation that began to bother him soon after his advent in the place he attributed to the drinking water, which, he thought, did not agree with him.

In spite of some treatment, his symptoms steadily grew worse during the fall and winter months, when he finally presented himself to Dr. Thos. McCann, who sounded him and discovered a stone in the bladder. He was sent to the West Penn Hospital, and I operated upon him as before stated.

No trouble was experienced at the time of the operation; there was some bleeding, but no more than is usually seen; the stone was extracted slowly, and the bladder carefully washed out. I saw the patient about half an hour after the operation, and, though I did not examine the bed, I felt the boy's pulse, and left him thinking all was well. About fifteen minutes after this the nurse noticed that he was bleeding freely. Dr. C. B. King, who happened to be at hand, introduced an umbrella-shaped tent into the wound, a catheter forming the center of the tent, and oil silk the outer, the space between the silk and the catheter being packed with cotton. This seemed to arrest the hemorrhage for a time, but the presence of the catheter and clots in the bladder produced irritation, constant straining, with expulsion of the tent, and a return of the hemorrhage.

Between three and four o'clock, some three hours after the operation, Dr King having gone home, I was summoned to the hospital, and on my arrival found my patient with head down and feet elevated; he was pale, sweating, vomiting, thirsty, with a rapid pulse, and in anything but a promising condition. I examined the wound, but was unable to find the source of hemorrhage. I removed some clots from the bladder, tried to introduce a fresh tent, but the attempt at introduction produced so much straining that I immediately abandoned it.

I then slipped a piece of gauze, well oiled, over my index finger, introduced it into the wound, and, withdrawing the finger, packed the space as well as possible with absorbent cotton; but this did not appear to arrest the hemorrhage, the oozing still continuing. The irritation of the bladder being so great as to preclude the introduction of anything within its walls, we concluded to flush the intestines with hot water, and see what effect that would have.

A flexible rectal tube was introduced well up to the sigmoid flexure of the colon, and a large quantity of hot water forced into the bowel. The rectal tube was withdrawn, a pad placed over the rectum and tent (which had been held in position) with a T bandage over all. I am happy to say that the effects of the hot water were soon made apparent, the pulse improved, and the hemorrhage was arrested.

At eight o'clock that night the patient had reacted ; there being odor of urine in the bed, the pads were not removed until the following morning, when they were discontinued. Dr. Todd, the resident in charge, kindly sat up with the boy the first night, giving him hypodermic injections of whisky or aromatic spirits of ammonia when required.

The patient, though weak for a time, made an uninterrupted recovery from this on, being absolutely free from pain. On the 28th of April he passed water through his uretha. On the 29th he passed a minute fragment of stone, evidently one of the small points which you will see is missing in the specimen.

The boy was discharged from the hospital perfectly well on the 15th of May, one month after the operation.

It is probable that the vessel from which the bleeding came was injured during the extraction of the stone, which is studded with sharp points, as you see, and that it gave way at the time or a short time after the patient was lifted into bed.

DR. BUCHANAN: It was my fortune six years ago to assist Dr. Kersey in a similar case. At St. Francis Hospital he removed a very large stone through an incision which was probably of necessity insufficient. At all events, there was a great deal of trouble in removing the stone, and, although there was no hemorrhage at the time, there must have been considerable contusion of the tissues. If I recollect aright on the sixth day a very severe hemorrhage came on. Dr. Kersey was sent for late in the afternoon, and I went with him to assist in checking the bleeding. We tried all the ordinary means to stop the hemorrhage, endeavoring first, of course, to secure the bleeding vessel. The man seemed to be at the point of death, and we were not long in trying the umbrella catheter ; but in this instance, at least, it was a dismal failure. A sufficient pressure could not be exerted. We then passed a catheter of considerable calibre as far as the bladder, and put two or three sponge-tents along-side of it. The bleeding was instantly checked, and the man recovered.

DR. STEWART : I have had two caseses—one hemorrhage from rupture of the urethra ; there had been several attacks of hemorrhage, the patient becoming very faint. When I saw him he was bleeding, and there was an opening in the perineum, which had been made to relieve the extravasation. The hemorrhage was checked by plugging around a silver catheter placed in the perineal opening. The second case was similar to the first, except that the hemorrhage was caused by ulceration from prolonged pressure of a silver catheter against the margins of an exploratory perineal opening. The hemorrhage was checked in similar manner to the preceding case.

